# Normal serum uric acid gout: a neglected and challenging condition

**DOI:** 10.3389/fendo.2026.1873856

**Published:** 2026-06-11

**Authors:** Xiu Hong Yang, Xiao Li Zhan, Qing Xia Xuan, Hui Min Jin, Zhi Bin Ye

**Affiliations:** 1 Department of Nephrology, Huadong Hospital, Fudan University, Shanghai, China; 2 Shanghai Key Laboratory of Clinical Geriatric Medicine, Huadong Hospital, Fudan University, Shanghai, China; 3 Department of Nephrology, Shidong Hospital affiliated to University of Shanghai for Science and Technology, Shanghai, China; 4 Shanghai Dong Ji Fresenius Hemodialysis Center, Shanghai, China; 5 Division of Nephrology, Shanghai Pudong Hospital, Fudan University, Shanghai, China

**Keywords:** diagnostic imaging, gout, hyperuricemia, monosodium urate crystals, uric acid

## Abstract

During acute gout flares, serum uric acid (SUA) levels may remain within the normal range, making diagnosis based solely on SUA unreliable. A systematic evaluation incorporating clinical symptoms and imaging (ultrasound, dual-source CT) is essential to detect monosodium urate (MSU) crystal deposition. Normal-SUA gout often presents with more pronounced inflammation, fever, elevated white blood cell count and C-reactive protein, and may occur after surgery or hemodialysis. Tophi can appear at typical or atypical sites (e.g., face, neck). No specific treatment guidelines exist, but lowering SUA to <360 μmol/L (6 mg/dl) reduces gout flares and promotes MSU dissolution. Emerging therapies—including anti-IL-1β monoclonal antibodies, NLRP3 inhibitors, uricases, and green tea components—show promise in controlling attacks and dissolving crystals without affecting SUA levels.

## Introduction

Gout is one of the most common inflammatory arthritides worldwide, resulting from the deposition of monosodium urate (MSU) crystals in joints, bones, and soft tissues. These crystals trigger recurrent, self-limited inflammatory flares that can cause severe pain, swelling, redness, and functional impairment, significantly reducing patients’ quality of life (1). The global prevalence of gout ranges from 1% to 4%, with a striking male predominance (male-to-female ratio 3–10:1). The prevalence increases sharply with age, affecting up to 11–13% of individuals over 80 years (2). Certain ethnic groups, including US minorities, Māori, Han Chinese, and other Asian populations, bear a disproportionately high burden, suggesting both genetic and environmental contributions to disease susceptibility (2).

In this review, “normal serum uric acid (SUA)” is defined according to American College of Rheumatology (ACR) guidelines as <360 μmol/L for women and <420 μmol/L for men. Hyperuricemia, defined as serum uric acid (SUA) ≥ 360 μmol/L in women and ≥ 420 μmol/L in men, has historically been considered the central biochemical abnormality and diagnostic cornerstone for gout (3, 4). Indeed, the risk of incident gout increases progressively with rising SUA levels, and longstanding hyperuricemia is the most important risk factor for MSU crystal formation. However, it is now well established from both clinical experience and prospective cohort studies that acute gouty arthritis can occur when SUA is within normal or even low. This phenomenon—herein referred to as normal-SUA gout—is frequently under-recognized in clinical practice, leading to delayed diagnosis, unnecessary diagnostic procedures, inappropriate use of antibiotics or other anti-inflammatory agents, and prolonged patient suffering (5–13). These pronounced inflammatory features can mimic infection, sometimes leading to inappropriate systemic antibiotic therapy (5).

Several factors contribute to the under-diagnosis of normal-SUA gout. First, many clinicians adhere to the intuitive but incorrect notion that a normal SUA effectively excludes gout. This misconception is reinforced by traditional teaching and many textbook descriptions that emphasize hyperuricemia as a diagnostic prerequisite. Second, the inflammatory response during an acute flare can paradoxically lower SUA, masking an underlying chronic hyperuricemia. Third, even in the absence of systemic hyperuricemia, local factors within the joint microenvironment—including temperature, pH, ionic composition, protein interactions, and connective tissue conditions—may favor MSU crystallization. Fourth, genetic variants affecting urate transporters such as SLC2A9 and ABCG2 can predispose individuals to MSU deposition despite normal circulating urate levels.

The advent of advanced noninvasive imaging techniques, particularly high-frequency ultrasound and dual-energy computed tomography (DECT), has revolutionized the diagnosis of gout in recent years. These tools allow direct visualization of MSU deposits in joints and soft tissues, regardless of SUA levels, and have greatly improved our ability to diagnose normal-SUA gout. However, their diagnostic performance in normal-SUA gout has not been systematically evaluated across diverse populations, and detection rates vary widely across studies (6). The variability in detection rates across studies underscores the need for a comprehensive review integrating epidemiology, imaging, and clinical features.

This review aims to provide a comprehensive, evidence-based overview of normal-SUA gout, covering its prevalence, pathogenic mechanisms, distinctive clinical features, diagnostic strategies, and current treatment approaches. By raising awareness of this neglected condition, we hope to improve diagnostic accuracy and clinical outcomes for affected patients.

## Methods

This article is a narrative review based on a structured literature search. We searched MEDLINE via PubMed, Web of Science, EMBASE, ClinicalTrials.gov, the Cochrane Central Register of Controlled Trials, and China National Knowledge Infrastructure for relevant publications on gout with normal serum uric acid. The search covered records from database inception to 2025, except for ClinicalTrials.gov, where the search was restricted to studies registered between 2000 and 2025. The complete database-specific search strategies are provided in **Supplementary Table 1**.

The search strategy combined controlled vocabulary terms, where applicable, with free-text keywords related to gout, serum uric acid or serum urate, normouricemia, monosodium urate crystals, imaging, ultrasound, and dual-energy computed tomography. Medical Subject Headings were used for MEDLINE via PubMed and Cochrane CENTRAL, Emtree terms were used for EMBASE, topic-field searches were used for Web of Science, and Chinese and English keywords were used for CNKI.

Two reviewers independently searched the literature and screened titles, abstracts, and full texts for relevance. Original clinical studies, cohort studies, case series, case reports, clinical trials, reviews, and mechanistic studies were considered if they provided information relevant to normal-SUA gout, MSU crystal deposition, imaging diagnosis, pathophysiology, or treatment. Articles were excluded if they were unrelated to gout, did not discuss serum uric acid or MSU crystal deposition, were not available in English or Chinese, or lacked relevance to the scope of this review. Disagreements between the two reviewers were resolved through discussion, and a third reviewer was consulted when consensus could not be reached.

### Prevalence of normal SUA during acute gout

The reported prevalence of normal SUA during acute gout attacks varies considerably across studies, ranging from as low as 11% to as high as 63.3% (7–16). This wide variation reflects differences in study populations (community-based vs. clinic-based vs. hospital-based), diagnostic criteria (clinical diagnosis based on American College of Rheumatology criteria versus crystal-confirmed gout), definitions of “normal” SUA (which may differ by sex and laboratory reference ranges, typically 360–420 μmol/L for men and 240–360 μmol/L for women), timing of SUA measurement relative to flare onset (during the first 24 hours versus later in the flare), and prior use of urate-lowering therapy or diuretics. **Table 1** summarizes ten key prospective and retrospective studies. The prevalence of normal-SUA gout varies widely (11%–63.3%), reflecting differences in diagnostic criteria, timing of SUA measurement, and study population characteristics. Crystal-proven diagnoses generally report lower prevalence, whereas clinical criteria or colchicine response yield higher rates. Early SUA measurement during flares often shows higher prevalence due to transient inflammation-induced reductions. Hospitalized or post-surgical patients also tend to have higher prevalence, likely reflecting more severe inflammation or metabolic changes. Overall, these differences reflect methodological and patient-specific factors rather than random variation. Several large prospective cohort studies have provided important insights into the relationship between baseline SUA and incident gout risk, even within the so-called normal range (17–19). In the Malmö Preventive Project, which followed 33,346 participants for up to 30 years, men with SUA levels between 360 and 405 μmol/L had a hazard ratio for incident gout of 4.4 (95% CI 2.9–6.7) compared to those with lower levels. For women, the corresponding hazard ratio was 4.7 (3.1–7.2) (17). These findings indicate that even modestly elevated urate levels within the normal range carry a substantially increased gout risk, and that the relationship between SUA and gout risk is continuous rather than threshold-based.

**Table 1:** Incidence of normal SUA in several cohort studies.

Study	Type of cohort (n)	Setting	Method of diagnosis	% with normal SUA
Malik A, et al., 2009 (7)	Prospective (28)	Veterans Administration rheumatology clinic	Crystal positivity	11%
Logan JA, et al., 1997 (8)	Prospective (38)	Multiple settings (eg, inpatient, clinic, ED)	Clinical criteria or crystal positivity	43%
Zhang J, et al.2023 (9)	Prospective (32)	Clinic patients	NA	31.25%
Zhao T, et al., 2018 (10)	Retrospective (126)	Outpatients	NA	34.92%
Park YB, et al., 2003 (11)	Retrospective (226)	Hospitalized patients	Clinical criteria or crystal positivity	12%
Schlesinger N, et al., 2009 (12)	Retrospective (339)	Multiple settings	Crystal positivity	32%
Urano W, et al., 2002 (13)	Retrospective (41)	Rheumatology clinic	Clinical criteria	49%
Hall AP, et al., 1967 (14)	Retrospective (69)	Multiple settings	Clinical criteria	33%
Bădulescu M, et al.2014 (15)	Retrospective (30)	Hospitalized patients	clinical manifestations and positive response to therapeutic test with colchicine	63.3%
Lee JS, et al., 2020 (16)	Retrospective (221)	Multiple settings	Crystal positivity	39.8%

Prevalence of normal-SUA gout varies across studies (11%–63.3%) due to differences in diagnostic criteria, timing of SUA measurement, and patient characteristics, with higher rates observed in clinically diagnosed or early-measured flares and in hospitalized or post-surgical patients. SUA, serum uric acid; ED, emergency department; NA, not available. Prevalence values are shown with 95% confidence intervals where reported.

Another prospective study pooling individual participant data from 18,889 individuals demonstrated a remarkable 15-year cumulative incidence of gout, ranging from just 1.1% among participants with SUA <360 μmol/L to 49% among those with SUA ≥600 μmol/L (18). A longitudinal cohort study further confirmed that gout incidence increases progressively with baseline SUA, from 0.59% at <240 μmol/L to 12.2% at >420 μmol/L (19). Together, these epidemiological data firmly establish that while hyperuricemia is a major risk factor, gout can and does occur across the full spectrum of SUA levels, including those conventionally considered normal. Therefore, a normal SUA should never be used alone to exclude a diagnosis of gout in a clinically suggestive case. Instead, SUA should be interpreted in the context of the patient’s clinical presentation, flare timing, and risk factors.

### Mechanisms of normal-SUA gout attacks

Understanding why acute gout can occur despite normal systemic SUA requires a multifactorial perspective that integrates inflammatory physiology, local joint biology, and genetic predisposition. Several interconnected mechanisms have been proposed and are supported by experimental and clinical evidence.

### Inflammatory reduction of SUA during acute flares

Perhaps the most important and best-documented mechanism is the paradoxical decline in SUA that occurs during acute gouty inflammation. Several studies have shown that patients with known hyperuricemia often experience a significant drop in SUA at the time of an acute flare, with levels returning to baseline after the flare resolves (8, 20, 21). This phenomenon is driven by increased renal uric acid excretion, mediated by inflammatory cytokines such as IL-1β and IL-6, which upregulate urate transporters (e.g., *URAT1, GLUT9, ABCG2*) in the proximal tubule (9, 10, 13). More recent evidence has extended this concept to the intestine: during acute gout attacks, intestinal uric acid excretion also increases, and this increase correlates with serum IL-1β levels (22). From an evolutionary perspective, enhanced uric acid excretion during inflammation may be a protective mechanism designed to limit MSU crystal burden. However, in clinical practice, it effectively masks the underlying hyperuricemia, leading clinicians to incorrectly conclude that gout is unlikely. Importantly, the magnitude of SUA reduction correlates with the intensity of inflammation, as measured by CRP and IL-6 levels, explaining why patients with more severe flares are more likely to have normal SUA (13).

### Local factors that promote MSU crystallization

Even when systemic SUA is within the normal range, the local microenvironment within joints and soft tissues can favor MSU crystal formation or persistence. Several factors contribute:

#### Temperature

Peripheral joints, particularly the first metatarsophalangeal (MTP) joint, are several degrees cooler than core body temperature (approximately 32–34 °C versus 37 °C). Since urate solubility decreases with falling temperature, crystal formation can occur even at normal systemic urate concentrations. This explains why the first MTP joint is the most common site of initial gout attacks (23, 24).

#### pH

Acidic conditions reduce urate solubility. Inflamed or traumatized tissues often become locally acidic due to lactic acid production and impaired perfusion, further promoting crystallization. Synovial fluid pH in inflamed joints can drop to 6.5–7.0, well below the physiological pH of 7.4 (23).

#### Ionic composition and proteins

Calcium, sodium, and other ions, as well as certain proteins (e.g., albumin, immunoglobulins, proteoglycans), can influence the nucleation and growth of MSU crystals. Some proteins act as promoters, while others (such as certain lipoproteins) may inhibit crystallization (23).

#### Connective tissue conditions

Fibrin, collagen, and other extracellular matrix components can serve as niduses for crystal deposition, providing a scaffold for crystal growth over time (23).

#### Secondary nucleation

Existing MSU crystals can seed the formation of new crystals, leading to propagation of crystal deposits even in the absence of ongoing supersaturation (23, 24).

Surgery, trauma, rapid weight loss, or rapid changes in urate metabolism (e.g., initiation of urate-lowering therapy, hemodialysis, administration of contrast agents) can acutely alter local pH, temperature, and protein composition, lowering the threshold for MSU precipitation and triggering a flare (25, 26). For example, postoperative gout flares are well recognized in patients undergoing bariatric surgery, joint replacement, or cardiac surgery, often occurring despite normal or even low SUA.

### Genetic susceptibility

Twin and family studies have long suggested a strong genetic component to gout, with heritability estimates of approximately 40–65%. Over the past decade, genome-wide association studies have identified multiple loci associated with gout risk, many of which encode urate transporters. The most extensively studied are *SLC2A9* (encoding GLUT9, a urate efflux transporter expressed in the kidney and intestine) and *ABCG2* (encoding a high-capacity urate exporter, also known as BCRP). Genetic variants in these transporters can impair renal or intestinal urate excretion, leading to elevated local urate concentrations in joints or soft tissues. As a result, MSU crystal deposition may occur even when circulating SUA remains within the normal range, explaining why individuals with “high-risk” genotypes can develop gout despite normal systemic urate levels (27, 28). For example, certain *ABCG2* variants (e.g., Q141K) are associated with a 2- to 4-fold increased risk of gout at any given SUA level, suggesting that tissue urate levels may be considerably higher than systemic concentrations due to impaired local excretion. Other genes, such as *SLC22A12* (URAT1) and *SLC17A1* (NPT1), also contribute to urate handling and gout risk.

### NLRP3 inflammasome activation

Once MSU crystals are deposited in joints or soft tissues, they are recognized by pattern recognition receptors, particularly the NLRP3 inflammasome. Activation of the NLRP3 inflammasome leads to cleavage and activation of caspase-1, which in turn processes pro-IL-1β and pro-IL-18 into their active, secreted forms. IL-1β is a master pro-inflammatory cytokine that recruits neutrophils, amplifies the inflammatory response, and accounts for the intense pain, swelling, and redness characteristic of acute gout. Importantly, IL-1β also feeds back to enhance uric acid excretion (both renal and intestinal), further lowering SUA and perpetuating the diagnostic challenge. This self-amplifying inflammatory loop explains why gout flares are often severe and self-limited: the inflammatory response promotes crystal clearance but also lowers SUA, potentially terminating the flare once crystals are sufficiently cleared (13, 22).

**Figure 1:**
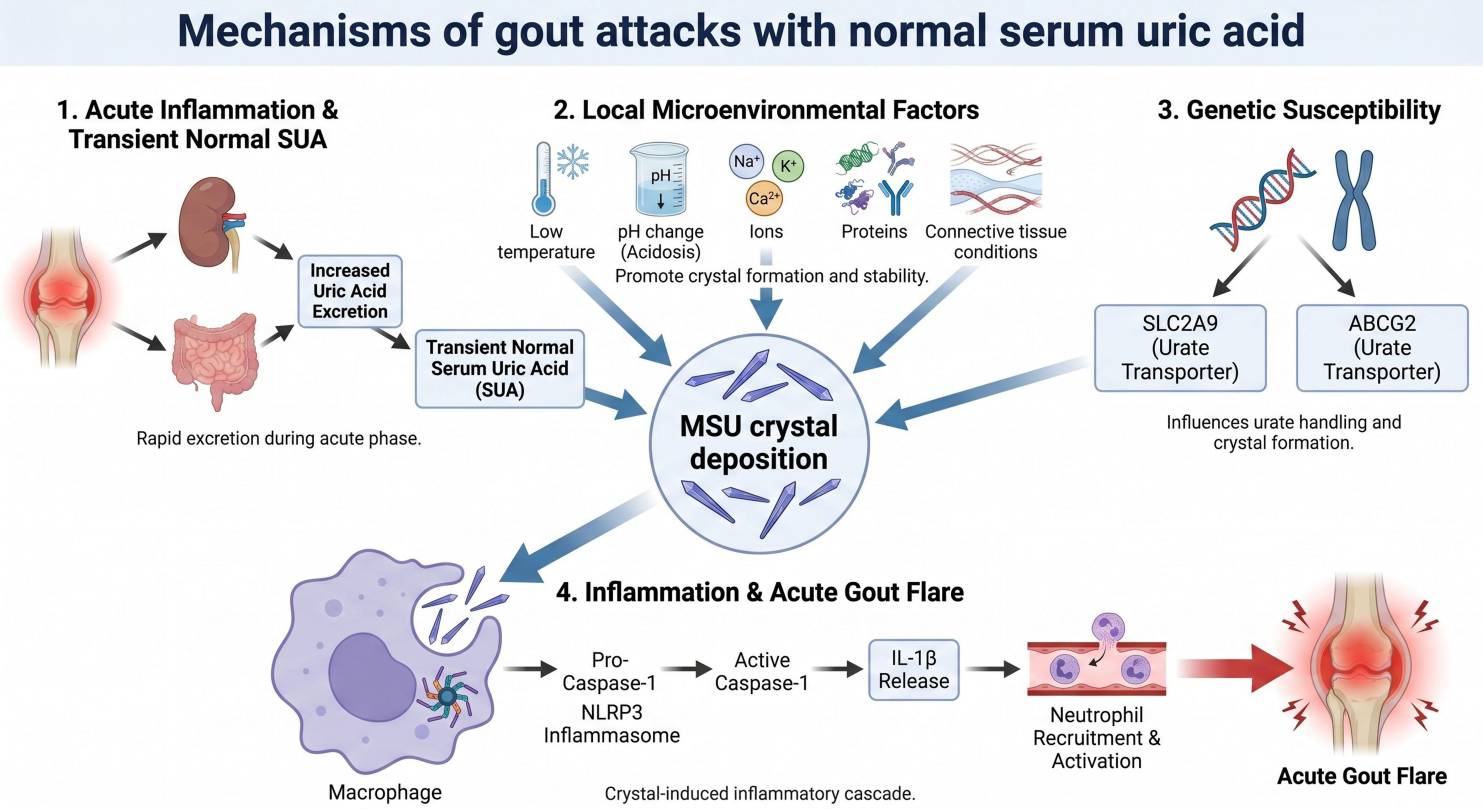
Mechanisms underlying normal-SUA gout attacks. Acute gout flares may occur despite normal circulating SUA due to: (1) enhanced renal and intestinal uric acid excretion triggered by inflammation, (2) local microenvironmental factors promoting MSU crystallization (temperature, pH, ions, proteins, connective tissue), and (3) genetic predisposition affecting urate transport (SLC2A9, ABCG2). Deposited MSU crystals activate the NLRP3 inflammasome, inducing IL-1β-mediated inflammatory cascades that drive pain, swelling, and systemic inflammatory responses. SUA, serum uric acid; MSU, monosodium urate; IL-1β, interleukin-1 beta.

A proposed mechanistic model integrating these pathways—inflammatory urate excretion, local crystallization factors, genetic susceptibility, and inflammasome activation—is shown in **Figure 1**.

### Clinical characteristics

The clinical presentation of normal-SUA gout shares many features with classic hyperuricemic gout, but several distinctive characteristics have been identified that may aid in recognition and differential diagnosis.

### Joint involvement

The distribution of affected joints in normal-SUA gout is broadly similar to that of hyperuricemic gout. The first metatarsophalangeal joint (big toe) remains the most commonly affected site, involved in approximately 50–70% of first attacks. The ankle, knee, fingers, wrist, and elbow are also frequently involved. Oligoarticular involvement (two or three joints) occurs in about 30–40% of attacks, and polyarticular involvement (four or more joints) can occur, particularly in patients with longer disease duration, older age, or underlying comorbidities such as chronic kidney disease or diabetes. Lower extremity joints are more commonly affected than upper extremity joints, consistent with the temperature-dependent solubility of urate (29).

### Systemic inflammation

Several studies have reported that patients with normal-SUA gout exhibit more intense systemic inflammation than their hyperuricemic counterparts. Specifically, they are more likely to present with fever (temperatures >38 °C), higher white blood cell counts (often >12,000–15,000/μL), and more markedly elevated C-reactive protein (CRP) levels (frequently >50–100 mg/L) (16). This heightened inflammatory response may be related to the rapidity of crystal shedding (a “shower” of crystals rather than gradual deposition), the absence of chronic immunosuppressive adaptation that occurs in longstanding hyperuricemia, or differences in the inflammatory cytokine milieu (e.g., higher IL-1β, IL-6, or TNF-α levels). Clinicians should be aware that such pronounced systemic inflammation can mimic septic arthritis or other serious infections, and appropriate diagnostic evaluations (including joint aspiration and culture) are necessary to exclude infection before initiating anti-inflammatory therapy.

### Precipitating factors

A history of recent surgery (within days to weeks) or maintenance hemodialysis is more common in patients with normal-SUA gout compared to those with hyperuricemic gout (16). Surgical procedures, particularly orthopedic, bariatric, and cardiac surgeries, can induce local tissue injury, fluid shifts, rapid weight loss, and transient changes in urate metabolism that promote crystal shedding. In hemodialysis patients, rapid removal of urate during dialysis can similarly destabilize existing MSU deposits, triggering an acute flare. Other recognized precipitants include initiation of urate-lowering therapy (so-called “paradoxical flares”), rapid weight loss (including after bariatric surgery), dehydration, fasting, and administration of medications that affect urate levels (e.g., loop diuretics, low-dose aspirin, cyclosporine). Recognition of these precipitating factors should raise clinical suspicion for normal-SUA gout in the appropriate setting.

### Tophus deposition

Tophi are aggregates of MSU crystals surrounded by a chronic granulomatous inflammatory response, often with fibrosis and foreign-body giant cells. While tophi are typically associated with long-standing, poorly controlled hyperuricemia (usually >10 years of disease), they can also occur in patients with normal SUA, particularly those with a strong genetic predisposition or repeated episodes of acute inflammation without sustained hyperuricemia. Tophus locations include typical sites such as the olecranon bursa, Achilles tendon, patellar tendon, fingers (especially the interphalangeal joints), wrists, and elbows, as well as atypical sites such as the nasal dorsum, auricle (ear helix), head and neck, vocal cords, spine, and even the breasts (30–33). Atypical tophi frequently cause diagnostic confusion and may be biopsied for suspected malignancy, rheumatoid nodule, or other inflammatory conditions. The presence of tophi in a patient with normal SUA should prompt consideration of gout rather than exclusion based on urate levels. Imaging, particularly DECT, can be invaluable in confirming the diagnosis noninvasively.

### Family history

Family history is a strong risk factor for gout, independent of SUA levels (34). The Sons of Gout Study provided compelling evidence for familial aggregation of MSU deposition independent of SUA levels. In this study, 24.2% of asymptomatic sons of patients with gout had ultrasound-detected MSU deposition when their own SUA levels were between 5 and 6 mg/dl (approximately 300–360 μmol/L), compared to only 6.7% of sons of controls (34). This finding demonstrates that normal SUA does not preclude crystal formation and that imaging-detected MSU deposits may be a more sensitive marker of gout risk than SUA measurement alone. Clinicians should therefore inquire about family history of gout in any patient presenting with acute arthritis, even if SUA is normal.

### Diagnostic methods

#### Gold standard

The definitive diagnosis of gout rests on the identification of MSU crystals in synovial fluid aspirated from an inflamed joint or in material obtained from a tophus. Under polarized light microscopy, MSU crystals appear as needle-shaped, negatively birefringent crystals (yellow when parallel to the compensator axis, blue when perpendicular). The sensitivity of polarized light microscopy for gout is high (approximately 85–95%) when performed by experienced observers, and specificity approaches 100%. However, joint aspiration is invasive, requires technical expertise, may be difficult to perform in certain joints (e.g., hip, sacroiliac joints, small joints of the hands and feet), and is often deferred in the emergency department or primary care setting. Moreover, crystal examination is operator-dependent and can yield false-negative results if the aspirate is inadequate, if crystals are sparse, if the sample is not examined promptly (crystals can dissolve over time), or if the patient has received colchicine or NSAIDs prior to aspiration (35, 36).

### Noninvasive imaging

For these reasons, noninvasive imaging has become an indispensable tool for diagnosing gout, particularly in normal-SUA cases where clinical suspicion is high but biochemical confirmation is lacking.

Ultrasound: High-frequency ultrasound (≥10 MHz transducer) can detect several abnormalities indicative of MSU deposition, including:

- The double-contour sign: a hyperechoic, irregular band of MSU crystals deposited on the surface of hyaline cartilage, which appears as a second echogenic line parallel to the subchondral bone. This sign is highly specific for gout (approaching 95–100%) but has variable sensitivity (40–80%).
- Tophi: heterogeneous hyperechoic or hypoechoic aggregates with or without posterior acoustic shadowing, often surrounded by an anechoic halo (inflammatory tissue). Tophi can be measured longitudinally to monitor response to therapy.
- Erosions: cortical bone defects at joint margins, often with overhanging edges, representing chronic tophaceous erosion.
- Intra-articular aggregates (“snowstorm sign”): punctate hyperechoic foci within synovial fluid, representing free MSU crystals.

Ultrasound is widely available, noninvasive, well-tolerated, and can be performed at the bedside. It is also less expensive than CT or MRI. In a study of 38 patients with normal-SUA gout, 73.7% had involvement of at least two joint areas on ultrasound, a proportion similar to that observed in hyperuricemic controls (82.4%) (37). Another small study (n=12) found double-track sign, snowstorm sign, and synovial thickening as common ultrasound findings (38). The main limitations of ultrasound are operator dependence, the need for specialized training, and limited ability to assess deep or axial joints.

Dual-energy computed tomography (DECT): DECT uses two different X-ray energy spectra (typically 80 kVp and 140 kVp) to differentiate materials based on their atomic composition. MSU crystals are color-coded green on DECT, allowing direct visualization of crystal deposits regardless of SUA levels. DECT is particularly useful for:

- Detecting subclinical crystal deposition in patients with normal SUA.
- Assessing the extent (volume) of tophaceous deposits.
- Monitoring tophus burden over time in response to urate-lowering therapy.
- Identifying crystal deposits in deep or anatomically complex regions (e.g., spine, sacroiliac joints, hip).

DECT has high specificity for gout (approaching 95–100%) and moderate to high sensitivity (70–90% in most studies). Across seven Chinese studies, DECT detection rates in normal-SUA gout ranged from 66.7% to 92.3% (39–45), although one study reported a much lower rate of 28.6% (46). In a separate study of 224 patients with acute arthritis and low SUA, DECT identified MSU deposits in 12.4% (47). The variability in detection rates likely reflects differences in scanner generation, acquisition protocols, post-processing algorithms, patient characteristics (e.g., tophus burden, disease duration), and the proportion of patients with acute versus chronic gout. Large, prospective, multicenter studies in diverse populations are needed to establish standardized DECT protocols and interpretative criteria for normal-SUA gout.

Other imaging modalities: Conventional radiography is insensitive for early gout, typically showing only soft tissue swelling and normal bone density. Late findings include well-defined “punched-out” erosions with overhanging edges and sclerotic margins, but these take years to develop. MRI and CT without DECT capability are nonspecific and not recommended as first-line imaging for gout diagnosis.

It is important to recognize the limitations of ultrasound and DECT, especially in early-stage gout or first acute flares with normal SUA. Both modalities can yield false negatives when MSU deposits are small or sparse (35–37, 39). Ultrasound detection depends on crystal size and operator skill, while DECT sensitivity is reduced with minimal deposits or suboptimal imaging (35, 37, 39). Misdiagnosis is also a concern, particularly in acute monoarthritis, where septic arthritis can mimic gout (7, 30). Over-reliance on imaging alone may lead to over-diagnosis, especially in early-stage or asymptomatic crystal deposition. Clinicians should interpret imaging results alongside clinical presentation and laboratory findings, as a normal scan does not exclude gout.

### Differential diagnosis

Important differential diagnoses for acute arthritis in the setting of normal SUA include:

- Septic arthritis (requires urgent joint aspiration and culture; Gram stain and culture are essential).
- Calcium pyrophosphate deposition disease (pseudogout): crystals are rhomboid-shaped and weakly positively birefringent.
- Rheumatoid arthritis (usually chronic, symmetric, with morning stiffness and elevated rheumatoid factor/anti-CCP).
- Psoriatic arthritis (often asymmetric, with dactylitis, nail changes, and skin psoriasis).
- Reactive arthritis (typically following genitourinary or gastrointestinal infection, with asymmetric oligoarthritis and extra-articular features).
- Palindromic rheumatism (recurrent, self-limited episodes of mono/oligoarthritis with complete resolution between attacks).
- Hemarthrosis (trauma, anticoagulation, bleeding disorders).

**Figure 2:**
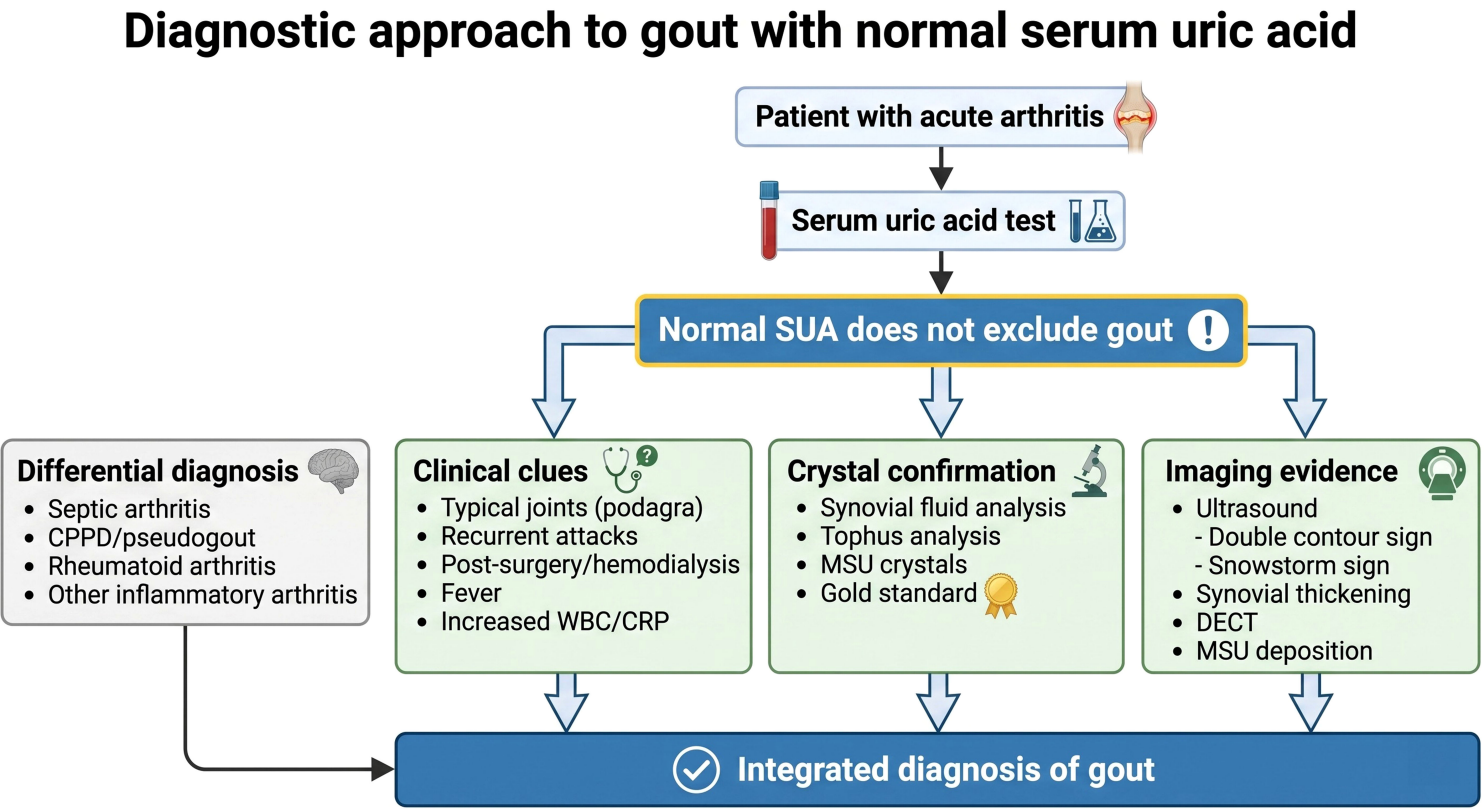
Diagnostic approach for gout with normal SUA. A normal SUA does not exclude gout in patients presenting with acute arthritis. Diagnosis should be guided by: pattern of joint involvement, recurrent attacks, systemic inflammatory features, and precipitating conditions (e.g., surgery, hemodialysis). Identification of MSU crystals in synovial fluid or tophus material remains the gold standard. When crystal confirmation is unavailable or impractical, US and DECT provide noninvasive evidence of MSU deposition. Differential diagnoses include septic arthritis, CPPD, “pseudogout”, RA, PsA, and other inflammatory arthritides. SUA, serum uric acid; MSU, monosodium urate; CPPD, calcium pyrophosphate deposition disease; RA, rheumatoid arthritis; PsA, psoriatic arthritis.

**Figure 3:**
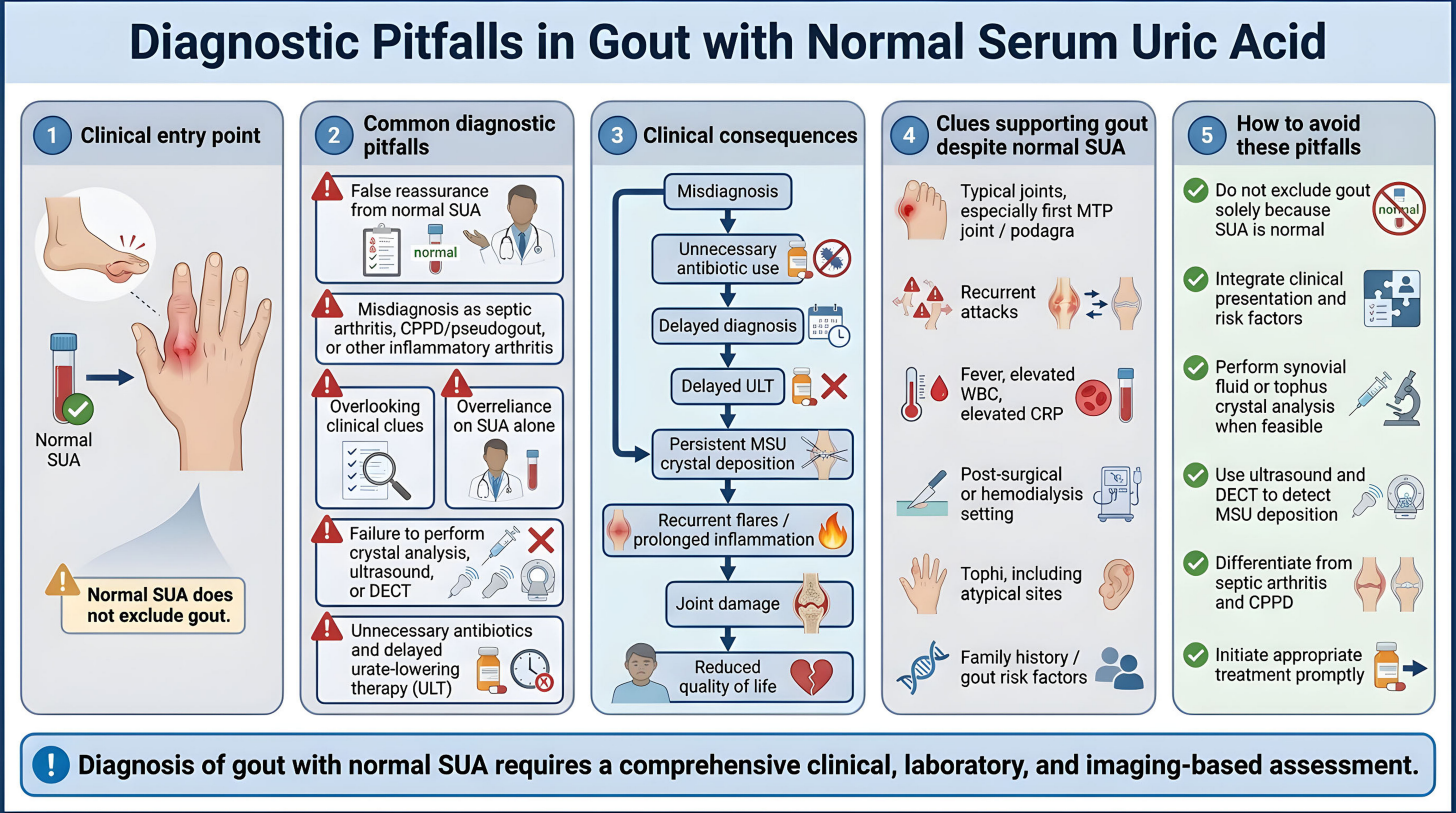
Diagnostic pitfalls in gout with normal SUA. This figure summarizes key challenges in diagnosing gout when SUA is normal, including misdiagnosis, unnecessary antibiotic use, and delayed ULT. Clinical clues such as typical joint involvement, recurrent attacks, fever, elevated WBC or CRP, post-surgical or hemodialysis setting, and tophi (including atypical sites) are highlighted. Strategies to avoid pitfalls include crystal confirmation, imaging (US, DECT), and timely initiation of ULT. SUA, serum uric acid; ULT, urate-lowering therapy; WBC, white blood cell count; CRP, C-reactive protein; MSU, monosodium urate; US, ultrasonography; DECT, dual-energy computed tomography.

A practical, stepwise diagnostic algorithm for normal-SUA gout is proposed in **Figure 2**. **Figure 3** illustrates key diagnostic pitfalls and strategies in gout patients with normal SUA.

### Health system implications

Normal-SUA gout challenges healthcare systems due to frequent misdiagnosis, inappropriate antibiotic use, and delays in urate-lowering therapy (ULT) (5, 16). Because SUA may remain normal during acute flares, clinicians can mistakenly exclude gout, leading to misattributed symptoms and unnecessary antibiotics (22). Delayed diagnosis also postpones ULT, allowing persistent MSU crystal deposition and prolonged inflammation, which worsens joint damage and patient quality of life (16, 35–37). Awareness of normal SUA gout and comprehensive evaluation—including clinical assessment, imaging, and crystal identification when possible—can reduce misdiagnoses, inappropriate treatments, and expedite effective therapy (35–37).

### Treatment strategies

#### Acute flare treatment

The management of acute flares in normal-SUA gout follows established principles for gout treatment in general, with the goal of rapid, effective inflammation control while minimizing adverse effects (48, 49).

#### Low-dose colchicine

Low-dose colchicine remains first-line therapy if initiated within 24–36 hours of flare onset. The recommended dosing regimen is 1.0 mg–1.2 mg initially, followed by 0.5 mg–0.6 mg one hour later, with careful attention to renal function and potential drug–drug interactions (e.g., with statins, macrolides, cyclosporine, verapamil). Low-dose colchicine is as effective as higher doses and significantly less toxic (reducing gastrointestinal side effects from ~70% to ~20–30%). Colchicine should be avoided or dose-reduced in patients with estimated glomerular filtration rate (eGFR) <30–50 mL/min.

#### Nonsteroidal anti-inflammatory drugs

Nonsteroidal anti-inflammatory drugs (NSAIDs) are an effective alternative, particularly in patients who cannot tolerate colchicine or in whom colchicine is contraindicated. High-dose NSAIDs (e.g., naproxen 500 mg twice daily, indomethacin 50 mg three times daily, ibuprofen 600–800 mg three times daily) for 5–7 days are typical. Caution is needed in patients with cardiovascular disease, chronic kidney disease, a history of gastrointestinal bleeding, or concurrent anticoagulant use. COX-2-selective inhibitors (e.g., celecoxib, etoricoxib) may have a lower risk of gastrointestinal bleeding but similar cardiovascular precautions apply.

#### Glucocorticoids

Glucocorticoids are indicated for patients with contraindications to both colchicine and NSAIDs, those with severe or polyarticular flares, those who have failed first-line therapies, or those with significant renal impairment. Options include oral prednisone (30–40 mg daily tapered over 7–10 days), intra-articular injection for monoarticular flares (e.g., triamcinolone 20–40 mg for large joints, 5–10 mg for small joints), or intramuscular triamcinolone (60 mg single dose). Glucocorticoids are highly effective but should be used cautiously in patients with diabetes, hypertension, or infectious risk.

#### Inter-critical and long-term urate-lowering therapy

Inter-critical and long-term urate-lowering therapy (ULT). The overarching goal of long-term management in gout—including normal-SUA gout—is to eliminate existing MSU deposits, prevent the formation of new crystals, reduce the frequency and severity of recurrent flares, and improve quality of life. Current international guidelines (EULAR, ACR, Japanese, Chinese) recommend maintaining SUA <360 μmol/L for most patients, with a lower target of <300 μmol/L for those with tophi or frequent flares (≥2 per year). SUA levels should generally not be lowered below 180 μmol/L (3 mg/dl) because of potential associations with neurodegenerative diseases (e.g., Parkinson’s disease, Alzheimer’s disease) and other adverse outcomes (50, 51).

For normal-SUA gout, there is no standardized ULT protocol, and treatment decisions must be individualized based on flare frequency, tophus burden, comorbidities, patient preferences, and the underlying SUA level (which may be in the high-normal range). However, a growing body of evidence supports the use of ULT in these patients. By reducing SUA to subsaturating levels (<360 μmol/L), ULT promotes the dissolution of existing MSU deposits (a process that typically takes 3–24 months depending on tophus burden) and prevents new crystal formation (52). In one prospective trial, febuxostat (a xanthine oxidase inhibitor) was administered to gout patients with near-normal SUA (<420 μmol/L). After one year of treatment, SUA levels were reduced to 180–300 μmol/L, tophus volume decreased by 62.8%, and gout flares were nearly eliminated (53). Comparable prospective data for allopurinol in normal-SUA gout are lacking, but it is reasonable to extrapolate from the extensive allopurinol experience in hyperuricemic gout, and clinical practice suggests similar efficacy.

When initiating ULT in normal-SUA gout, clinicians should be aware of the potential for “paradoxical” flare induction as MSU deposits begin to dissolve. Concomitant anti-inflammatory prophylaxis (e.g., low-dose colchicine 0.5–0.6 mg once or twice daily, or low-dose NSAIDs) for 3–6 months is recommended during the initial months of ULT, as per standard gout management guidelines. Standard treatments for acute flares—low-dose colchicine, NSAIDs, and glucocorticoids—and long-term urate-lowering therapy (allopurinol, febuxostat) are validated in humans.

Management of normal SUA gout remains challenging due to limited high-quality evidence. Randomized controlled trials specifically addressing this population are lacking, restricting evidence-based guidance. Clinicians must balance the potential benefits of ULT against the risk of overtreatment, particularly in patients with infrequent flares or minimal joint involvement. ULT may be appropriate for patients with recurrent flares, tophi, or radiographic evidence of joint damage, but routine use in all normal-SUA patients is not supported. Shared decision-making considering flare frequency, comorbidities, and patient preferences is essential to guide therapy (50, 51).

### Emerging therapies

Several novel therapeutic approaches hold particular promise for normal-SUA gout because they target the inflammatory response or promote crystal dissolution without necessarily lowering SUA. These may be especially valuable for patients who cannot tolerate or have contraindications to standard ULT (54–58). Pegloticase, approved for refractory tophaceous gout, is reserved for severe cases (59). Their clinical utility in normal SUA gout remains investigational.

#### Anti-IL-1β monoclonal antibody

Anti-IL-1β monoclonal antibody (canakinumab): In a large exploratory analysis of the CANTOS trial (n=10,059 participants with prior myocardial infarction and high-sensitivity CRP ≥2 mg/L; median SUA 362.9 μmol/L), canakinumab (150 mg subcutaneously every 3 months) significantly reduced the rate of gout attacks across all baseline SUA levels. The hazard ratio for gout attacks was 0.36 (95% CI 0.24–0.54) compared to placebo. Importantly, this effect was observed without any change in SUA levels, making canakinumab uniquely suitable for patients with normal-SUA gout who cannot or should not receive traditional ULT (e.g., those with intolerance to allopurinol/febuxostat, or those with very frequent flares despite normal SUA) (54). However, canakinumab is expensive, requires subcutaneous injection, and carries risks of immunosuppression (increased risk of serious infections, including tuberculosis). It is currently approved for certain autoinflammatory diseases but not specifically for gout in most countries.

#### NLRP3 inflammasome inhibitors

NLRP3 inflammasome inhibitors: Preclinical studies have identified several small molecules that inhibit NLRP3 inflammasome activation. Caffeic acid phenethyl ester (CAPE), a component of propolis, and sulforaphane (SFN), a dietary isothiocyanate found in cruciferous vegetables (e.g., broccoli, Brussels sprouts), have been shown to suppress NLRP3 activation and IL-1β production in murine gout models, reducing joint inflammation and pain behaviors (55, 56). These agents are still in early development and have not yet been tested in human trials for gout, but they represent a promising strategy for treating acute gout without affecting urate metabolism.

#### Green tea polyphenol

**Green tea polyphenol:** Epigallocatechin-3-gallate (EGCG), the major catechin in green tea, inhibits NLRP3 inflammasome activation and mitochondrial DNA synthesis in macrophages, and reduces MSU-induced peritoneal inflammation in mice (57, 58). If confirmed in human trials, EGCG or its derivatives could offer a safe, accessible, and inexpensive adjunctive therapy for gout, possibly as a dietary supplement or functional food.

#### Recombinant uricases

Recombinant uricases (pegloticase): Uricases catalyze the conversion of poorly soluble uric acid to highly soluble allantoin, which is readily excreted by the kidneys. Pegloticase rapidly depletes both SUA and preformed MSU deposits. It is approved for refractory, chronic tophaceous gout in the United States and Europe, typically for patients who have failed or are intolerant to conventional ULT. In combination with methotrexate to reduce immunogenicity, pegloticase can achieve rapid tophus dissolution (within weeks to months) and even bone erosion remodeling as detected by DECT (59, 60). While most studies have focused on patients with severe hyperuricemia, there is no theoretical reason why pegloticase would not be effective in normal-SUA gout with significant tophus burden. However, its high cost (>$10,000–20,000 per month), intravenous administration (every 2–4 weeks), and immunogenicity (infusion reactions, loss of efficacy due to anti-drug antibodies) limit its use to carefully selected patients with severe, disabling, treatment-refractory gout.

**Figure 4:**
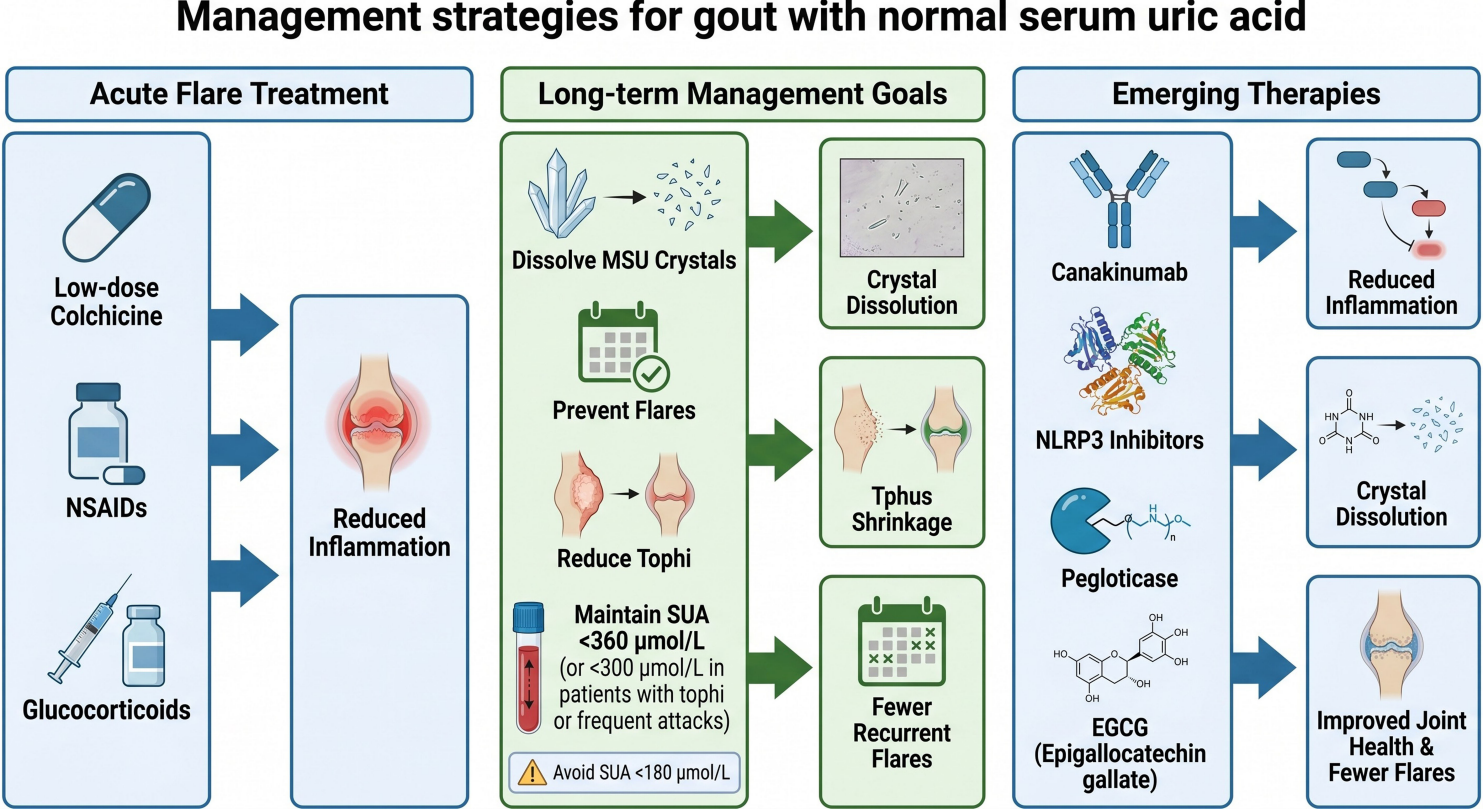
Management strategies for gout with normal SUA. Management integrates acute flare control and long-term reduction of MSU crystal burden. Acute flares are treated with low-dose colchicine, NSAIDs, or glucocorticoids, chosen based on severity, comorbidities, and contraindications. Long-term therapy aims to dissolve existing MSU deposits, prevent recurrence, and reduce tophus burden, targeting SUA <360 μmol/L (6 mg/dL) or <300 μmol/L (5 mg/dL) for patients with tophi or frequent flares. Excessive urate lowering should be avoided. Emerging options include anti−IL−1β monoclonal antibody (e.g., canakinumab), NLRP3 inflammasome inhibitors, uricase-based therapy (e.g., pegloticase), and EGCG supplementation. SUA, serum uric acid; MSU, monosodium urate; IL-1β, interleukin-1 beta; NSAID, nonsteroidal anti-inflammatory drug; EGCG, epigallocatechin-3-gal.

The overall management strategy for normal-SUA gout, including acute treatment, long-term urate control, and emerging therapies, is summarized in **Figure 4**.

## Limitations and recommendations

This review has some limitations. We conducted a narrative review rather than a meta-analysis, so we could not combine data across studies. The studies we included were quite different in design, patient populations, definitions of normal-SUA gout, and imaging methods, which makes direct comparisons difficult. Some evidence came from small or retrospective studies or case reports, so the overall certainty is limited.

For future research, we recommend using clear and consistent definitions of normal-SUA gout, reporting when SUA was measured relative to flare onset, and using crystal confirmation or reliable imaging like ultrasound or DECT. Large, prospective studies across multiple centers are needed. Clinically, physicians should not exclude gout just because SUA is normal; diagnosis should consider clinical features, risk factors, lab tests, crystal confirmation, and imaging. For patients with repeated flares, tophi, or imaging-confirmed MSU deposits, urate-lowering therapy and anti-inflammatory prophylaxis should be considered based on individual needs.

## Conclusion

Normal-SUA gout is not a rare clinical curiosity; rather, it is a commonly under-recognized and often misdiagnosed condition that affects a substantial proportion of patients with acute gouty arthritis. Reliance on SUA measurement alone as a diagnostic gatekeeper leads to missed or delayed diagnoses, inappropriate treatments, unnecessary patient suffering, and increased healthcare costs. Clinicians must recognize that a normal SUA does not exclude gout, particularly in patients with typical clinical presentations (e.g., acute monoarthritis of the first MTP joint or midfoot), characteristic risk factors (e.g., family history of gout, recent surgery, hemodialysis, rapid weight loss), or suggestive imaging findings (double-contour sign on ultrasound, green-coded MSU deposits on DECT).

The mechanisms underlying normal-SUA gout are multifactorial and include inflammation-induced enhancement of renal and intestinal uric acid excretion, local joint microenvironment factors (temperature, pH, ions, proteins) that promote MSU crystallization despite normal systemic urate levels, and genetic variants (e.g., *SLC2A9*, *ABCG2*) that alter urate transport and tissue deposition. These mechanisms are not mutually exclusive and may act synergistically in individual patients.

The diagnosis of normal-SUA gout should be based on a comprehensive assessment that integrates clinical presentation, risk factor analysis, and, when necessary, advanced imaging. Ultrasound and DECT are valuable noninvasive tools that can detect MSU deposits with high sensitivity and specificity, even in the absence of hyperuricemia. Invasive crystal confirmation remains the gold standard but is often not required in typical cases with supportive imaging findings.

Acute flares should be treated according to standard gout guidelines, with low-dose colchicine, NSAIDs, or glucocorticoids as appropriate, taking into account flare severity, comorbidities, and contraindications. For patients with recurrent flares, tophi, or radiographic evidence of MSU deposition, a trial of urate-lowering therapy targeting SUA <360 μmol/L (or <300 μmol/L in more severe cases) is reasonable and evidence-supported. Emerging therapies that target the NLRP3 inflammasome, IL-1β, or uricase offer exciting future possibilities, particularly for patients who cannot tolerate or do not respond to conventional ULT.

Ultimately, raising awareness of normal-SUA gout among internists, rheumatologists, emergency physicians, primary care providers, and even surgeons is an urgent priority. Educational initiatives, clinical decision support tools, and dissemination of diagnostic algorithms (such as **Figure 3**) may help improve recognition. Equally important is the conduct of well-designed, prospective, controlled studies in diverse populations to establish standardized diagnostic criteria, validate imaging protocols, optimize treatment algorithms, and explore the role of emerging therapies specifically for this neglected but challenging condition. Until then, clinical judgment and a high index of suspicion remain the most important tools for diagnosing and managing normal-SUA gout.
