# Supplementary material for: Normal serum uric acid gout: a neglected and challenging condition
Source: Front Endocrinol (Lausanne). 2026 Jun 11;17:1873856. doi: 10.3389/fendo.2026.1873856 (PMC13294634; doi:10.3389/fendo.2026.1873856)
Supplement: Supplementary file 1 [file Table1.docx]

**Supplementary Table 1: The search strategies of databases**

| **Databases** | **Search strategies** |
| --- | --- |
| **MEDLINE (PubMed)** | ("Gout"[Mesh] OR gout[Title/Abstract] OR gouty arthritis[Title/Abstract]) AND ("Uric Acid"[Mesh] OR serum uric acid[Title/Abstract] OR serum urate[Title/Abstract] OR normal serum uric acid[Title/Abstract] OR normal serum urate[Title/Abstract] OR normouricemia[Title/Abstract] OR normouricaemia[Title/Abstract]) AND ("monosodium urate"[Title/Abstract] OR "MSU crystals"[Title/Abstract] OR "urate crystal*"[Title/Abstract]) AND ("Diagnostic Imaging"[Mesh] OR "Ultrasonography"[Mesh] OR "Tomography, X-Ray Computed"[Mesh] OR imaging[Title/Abstract] OR ultrasound[Title/Abstract] OR ultrasonography[Title/Abstract] OR "dual-energy CT"[Title/Abstract] OR DECT[Title/Abstract]) |
| **Web of Science** | #1 TS=(gout OR “normal serum uric acid” OR normouricemia)  #2 TS=(“monosodium urate” OR “MSU crystals”)  #3 TS=(imaging OR ultrasound OR “dual-energy CT”)  #4 TS=(systematic review OR review)  #5 #1 AND #2 AND #3 AND #4 |
| **EMBASE** | #1 ‘gout’/exp  #2 ‘gout’:ab,ti OR ‘normal serum uric acid’:ab,ti OR ‘normouricemia’:ab,ti  #3 #1 OR #2  #4 ‘monosodium urate’/exp OR ‘MSU crystals’/exp  #5 ‘monosodium urate’:ab,ti OR ‘MSU crystals’:ab,ti  #6 #4 OR #5  #7 ‘imaging’/exp OR ‘ultrasound’/exp OR ‘dual-energy CT’/exp  #8 ‘imaging’:ab,ti OR ‘ultrasound’:ab,ti OR ‘dual-energy CT’:ab,ti  #9 #7 OR #8  #10 ‘systematic review’/exp OR ‘review’/exp  #11 ‘systematic review’:ab,ti OR ‘review’:ab,ti  #12 #3 AND #6 AND #9 AND (#10 OR #11) |
| **ClinicalTrials.gov** | Search for: (“gout” OR “normal serum uric acid” OR “normouricemia”) AND (“monosodium urate” OR “MSU crystals”) AND (“imaging” OR “ultrasound” OR “dual-energy CT”). Filter: Study type = “Interventional” or “Observational”; Status = “Completed” or “Recruiting”; Date: 2000–2025. |
| **Cochrane Central Register of Controlled Trials (CENTRAL)** | #1 MeSH descriptor: [Gout] explode all trees  #2 “normal serum uric acid” OR “normouricemia” in Title/Abstract  #3 “monosodium urate” OR “MSU crystals” in Title/Abstract #4 “imaging” OR “ultrasound” OR “dual-energy CT” in Title/Abstract  #5 #1 OR #2  #6 #3 AND #4  #7 #5 AND #6 |
| **China National Knowledge Infrastructure (CNKI)** | (痛风 OR gout) AND (血清尿酸 OR 尿酸 OR 正常血清尿酸 OR 正常尿酸 OR serum uric acid OR serum urate OR normouricemia) AND (尿酸单钠 OR 尿酸盐晶体 OR MSU晶体 OR monosodium urate OR MSU crystals) AND (影像学 OR 超声 OR 双能CT OR dual-energy CT OR DECT OR imaging OR ultrasound) |
